# Preparation and Characterization of Chitosan Nanoparticles for Chemotherapy of Melanoma Through Enhancing Tumor Penetration

**DOI:** 10.3389/fphar.2020.00317

**Published:** 2020-03-13

**Authors:** Hui Guo, Faping Li, Heping Qiu, Jianhua Liu, Sihao Qin, Yuchuan Hou, Chunxi Wang

**Affiliations:** Department of Urology, the First Hospital of Jilin University, Changchun, China

**Keywords:** chitosan, nanoparticle, penetrability, melanoma, chemotherapy

## Abstract

The poor solubility and permeability of most chemotherapeutic drugs lead to unsatisfactory bioavailability combined with insufficient drug concentration. In this study, positively charged nanoparticles based on chitosan were developed and synthesized to enhance tumor penetration capability of 10-Hydroxycamptothecin (HCPT) in order to improve the chemotherapeutic effect of melanoma. The HCPT encapsulated nanoparticles were noted as NPs/HCPT. NPs/HCPT was characterized by dynamic light scattering and *zeta* potential measurements. In addition, cell uptake, *in vitro* cytotoxicity, apoptosis and *in vivo* antitumor activity of NPs/HCPT were further investigated. The average diameter of NPs/HCPT was approximately 114.6 ± 4.1 nm. The viability of murine melanoma cell lines (B16F10 and B16F1) was significantly decreased due to interaction with NPs/HCPT. Moreover, NPs/HCPT significantly inhibited the progression of tumors. These investigations implied that cationic NPs/HCPT could be potentially applied as a promising drug delivery nanosystem.

## Introduction

Nowadays, one of the major causes of death all over the world is cancer due to its high heterogeneity (Bray et al., 2018). Chemotherapy is the preferred treatment for most cancer patients due to its universality and excellent efficacy (Al-Batran et al., 2019). Unfortunately, the poor solubility and permeability of most chemotherapeutic drugs lead to unsatisfactory bioavailability combined with insufficient drug concentration (Jia et al., 2018). Nanotechnology provides promising application prospects in encapsulating hydrophobic drugs, enhancing drug penetration depth, and targeting drug delivery (Fan et al., 2017). It has been reported that the physicochemical properties of nanoparticles, such as size, shape, and charge, are more meaningful to promote the accumulation of nanoparticles in tumor tissues than the surface modification of active targeting of nanoparticles (Danhier, 2016). Chitosan (CS) has been extensively investigated as an efficient drug delivery biopolymer due to its intriguing biocompatibility, biodegradability, anti-microbial activity, anti-inflammatory property, and nontoxicity (Nezakati et al., 2018; Zhao et al., 2018). This cationic polysaccharide is soluble in dilute acid and can adhere to the negatively charged biological membranes by electrostatic interaction, thus improving bioavailability and facilitating cell endocytosis (Kwak et al., 2019). In addition, CS has been reported to open the tight junction between epithelial cells, thereby enhancing the permeability of carried drugs (Sheng et al., 2015).

In this work, a positively charged drug delivery system based on CS was designed to enhance tumor penetration capability of 10-Hydroxycamptothecin (HCPT) in order to improve the chemotherapeutic effect of melanoma. As a broad-spectrum anticancer drug, HCPT is a commonly applied topoisomerase I inhibitor, which has promising antitumor property. The mechanism is mainly attributed to the effectively inhibition of DNA replication and RNA transcription in tumor cells (Guo et al., 2018; Li et al., 2018). HCPT was encapsulated into the core of nanoparticles (i.e., NPs/HCPT), which significantly improved the aqueous dispersibility and permeability. The positively charged NPs/HCPT could penetrate deep into the tumor and promote the internalization by tumor cells. The sustained release of HCPT from NPs/HCPT in the cytoplasm maintained an effective drug concentration, thus effectively inhibiting the proliferation of cancer cells. A comprehensive study was provided to further understand the application of NPs/HCPT in melanoma chemotherapy.

## Materials and Methods

### Materials

CS (degree of deacetylation of 80% and molecular weight of 50 kDa) was supplied by Sigma-Aldrich (Shanghai, P. R. China). 10-Hydroxycamptothecin (HCPT) was obtained from Beijing Huafeng United Technology Co., Ltd. (Beijing, P. R. China). Dulbecco's modified Eagle's medium (DMEM) and fetal bovine serum (FBS) were provided by Gibco (Grand Island, NY, USA). Penicillin and streptomycin were purchased from Huabei Pharmaceutical Co., Ltd. (Shijiazhuang, P. R. China). Trypsin and methyl thiazolyl tetrazolium (MTT) were supplied by Sigma-Aldrich (Shanghai, P. R. China). Annexin V-FITC and Propidium iodide (PI) were bought from Shanghai Qihai Futai Biotechnology Co., Ltd. (Shanghai, P. R. China). Acetic acid (HAc) and dimethyl sulphoxide (DMSO) were obtained from Shanghai Aladdin Bio-Chem Technology Co., Ltd. (Shanghai, P. R. China). N,N-dimethylformamide (DMF) was supplied by Sigma-Aldrich (Shanghai, P. R. China). The purified deionized water was produced by the Milli-Q plus system (Millipore Co., Billerica, MA, USA). All the other reagents were analytical grade and used as received without further purification.

### Cell Culture

Murine melanoma cell lines (B16F10 and B16F1) were obtained from Changchun Institute of Applied Chemistry (Jilin, China) and incubated in DMEM with 10% FBS, 100 μg ml^−1^ streptomycin and 100 μg ml^−1^ penicillin at 37°C in a 5% CO_2_ incubator.

### Preparation of HCPT Loaded Nanoparticles

The NPs/HCPT was fabricated according to the previous method with slight modification (Guo et al., 2018). Briefly, 25.0 mg of CS was prepared in 10.0 ml of HAc solution (pH 3.0), and then a solution of DMF containing 7.0 mg of HCPT was slowly added into the CS solution with stirring for 12 h at room temperature. The excess HCPT, which was not encapsulated inside the CS NPs, was removed by dialysis at room temperature. The whole process of purification was performed in darkness. Finally, NPs/HCPT was obtained by lyophilization.

### Degree of Drug Loading

To quantify the amount of HCPT entrapped in the prepared NPs, accurately measured volumes of freeze-dried NPs/HCPT were dissolved in HAc solution (pH 3.0). Consequently, the concentration of HCPT was assayed spectrophotometrically at wavelength of 371 nm using ultraviolet-visible (UV-vis). The drug loading content (DLC) and drug loading efficiency (DLE) of NPs/HCPT were expressed as percentages with respect to the standard curve method, and calculated according to the following equations.

(1) $$\text{DLC}(\%)=\frac{\text{Amount of drug in nanogel}}{\text{Amount of loading nanogel}}\times 100\%$$

(2) $$\text{DLE}(\%)=\frac{\text{Amount of drug in nanogel}}{\text{Total amount of feeding drug}}\times 100\%$$

### Particle Size and Zeta Potential Distribution

The particle size of NPs/HCPT was determined by dynamic light scattering (DLS) analyzer in a WyattQELS instrument with a vertically polarized He-Ne laser (DAWN EOS, Wyatt Technology Co., Santa Barbara, CA, USA). The concentration of NPs/HCPT for DLS was 100.0 μg ml^−1^. Before measurement, the samples were properly diluted in aqueous solution and filtered through a 0.45-μm filter. The *zeta* potential of NPs/HCPT was determined by a *zeta* potential analyzer (Brookhaven, USA). 100.0 μg ml^−1^ of NPs/HCPT dispersed in aqueous solution after sonication was detected as a sample. The dispersion was measured in triplicate and the results were reported as means values ± standard deviation (SD).

### In Vitro Drug Release Studies

The *in vitro* release profiles of HCPT from NPs/HCPT were performed in phosphate-buffered saline (PBS; pH 7.4, containing 0.1% Tween-80 (w/v)). Briefly, precisely weighed free HCPT (0.1 mg) or freeze-dried NPs/HCPT (1.0 mg) were suspended in 10.0 ml of release medium and then transferred into a dialysis cellulose bag (molecular weight cutoff (MWCO) = 3.5 kDa). The end-sealed dialysis bag was placed into a glass bottle containing 110.0 ml of release medium. The glass bottles were continuously shaken at 37°C. At experimental time intervals, aliquots of 2.0 ml were withdrawn and replenished with an equivalent amount of release medium. The samples were acidified with 1.0 N HCl, and analyzed by high-performance liquid chromatography (HPLC; λ_abs_ = 371 nm). The mobile phase consisted of 67:33 mix-tures of acetonitrile and aqueous buffer. The aqueous buffer was a mixture of 75 mmol L^−1^ ammonium acetate, 5 mmol L^−1^ triethylamine, and 0.5% (V/V) acetic acid. The flow rate was set at 1.0 ml/min.

### Cell Uptake and Distribution Studies

The cell uptake and intracellular distribution of NPs/HCPT were visualized by confocal laser scanning microscopy (CLSM). Typically, B16F10 cells were seeded into 6-well plates at a density of 2.0 × 10^5^ cells per well at 37°C containing 5% (v/v) carbon dioxide atmosphere. After 24 h of culture, the growth medium was discarded and the cells were incubated with either free HCPT or NPs/HCPT (HCPT concentration of 1.5 μg ml^−1^) for 2 h and 6 h. Afterward, the cells were carefully rinsed with PBS and fixed with 4% paraformaldehyde for 20 min at room temperature. At last, the obtained samples were washed with PBS for three times. The cell uptake and intracellular distribution of NPs/HCPT were confirmed by CLSM. In order to qualitatively understand the cell uptake of NPs/HCPT at different time points, B16F10 cells were cultured with either free HCPT or NPs/HCPT (HCPT concentration of 1.5 μg ml^−1^) for a consistent period of time, and then washed, lysed, dissolved, and then measured at 384 nm with a microplate reader (Tecan, Durham, USA). The experiment was repeated three times (Wei et al., 2010).

### In Vitro Cytotoxicity Assay

Cytotoxicity evaluation of NPs/HCPT was detected on melanoma cell lines (B16F10 and B16F1) by MTT assay. Briefly, B16F10 or B16F1 cells were seeded in 96-well plates at a density of 8.0 × 10^3^ cells per well. After 24 h, the cells were incubated with prepared solutions, including free HCPT and NPs/HCPT, for further 24 h or 48 h. The equivalent HCPT concentrations in culture medium ranged from 0 to 10.0 μg ml^−1^. After incubation period, 20.0 µl of MTT solution was added to each well and the cells were incubated at 37°C in 5% (v/v) CO_2_ atmosphere for approximately 4 h. Subsequently, the supernatant was removed and 200.0 µl of DMSO was added to dissolve the formazan crystals. The absorbance of obtained MTT-products was read using a Bio-Rad 680 microplate reader (Bio-Rad Laboratories, Hercules, CA, USA) at 490 nm. The untreated cells were used as a control. The cytotoxicity of empty NPs was studied on B16F1at the highest concentration of 20.0 μg ml^−1^. All experiments were performed in triplicate. The cell viability (%) was evaluated using the Equation 3 below.

(3) $$\text{Cell Viability}(\%)=\frac{A\text{sample}}{A\text{control}}\times 100\%$$

In Equation 3, *A*_sample_ and *A*_control_ referred to the absorbance of the sample and control well, respectively.

### Cell Apoptosis Analysis

The apoptosis percentage of B16F10 cells was evaluated by flow cytometry (FCM) analysis after treatment with NPs/HCPT for 24 h. Briefly, 3.0 × 10^5^ viable cells dispersed in 1.8 ml of DMEM medium were seeded in 6-well plates and allowed to attach overnight. Then 0.2 ml of growth medium containing different HCPT formulations was added with an equivalent HCPT concentration of 3.0 μg ml^−1^. The untreated cells were used as a control. After 24 h of incubation, the cells were harvested by trypsinization and collected by centrifugation at 1,000 rpm for 5 min. Subsequently, 0.5 ml of 1× Annexin V Binding Buffer was applied to resuscitate the obtained cells. Finally, the samples were double-labeled with Annexin V-FITC and propidium iodide (PI) on ice in the dark, and then analyzed by FCM.

### In Vivo Antitumor Activity Against Melanoma

Female C57BL/6 mice weighing 20–22 g were purchased from the Laboratory Animal Center of Jilin University. All animals received care in compliance with the guidelines outlined in the Guide for the Care and Use of Laboratory Animals, and all procedures were approved by the Animal Care and Use Committee of Jilin University. 1.0 × 10^5^ B16F10 cells dispersed in 0.1 ml of PBS were inoculated subcutaneously in the left flank of the mice. When the tumors reached approximately 50 mm^3^, mice were randomly divided into three groups: control, free HCPT, and NPs/HCPT (n = 5). Different HCPT formulations were intravenously administered to mice at a dose of 5.0 mg/kg every 3 days for four times. The body weight was also measured every 3 days during the process of the treatment.

### Statistical Analyses

The results were presented as means ± SD of three replicate independent experiments. Statistical analysis was performed using Student's t-test. *P* < 0.05 was considered statistically significance.

## Results and Discussion

### HCPT Encapsulation and NPs/HCPT Characterizations

**Figure 1:**
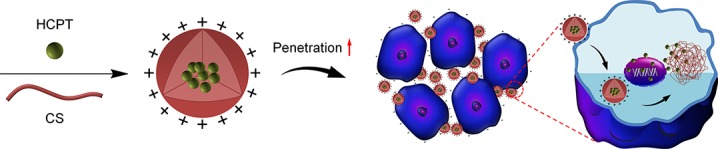
Schematic design of preparation and enhancement on the permeability of NPs/10-Hydroxycamptothecin (HCPT).

**Figure 2:**
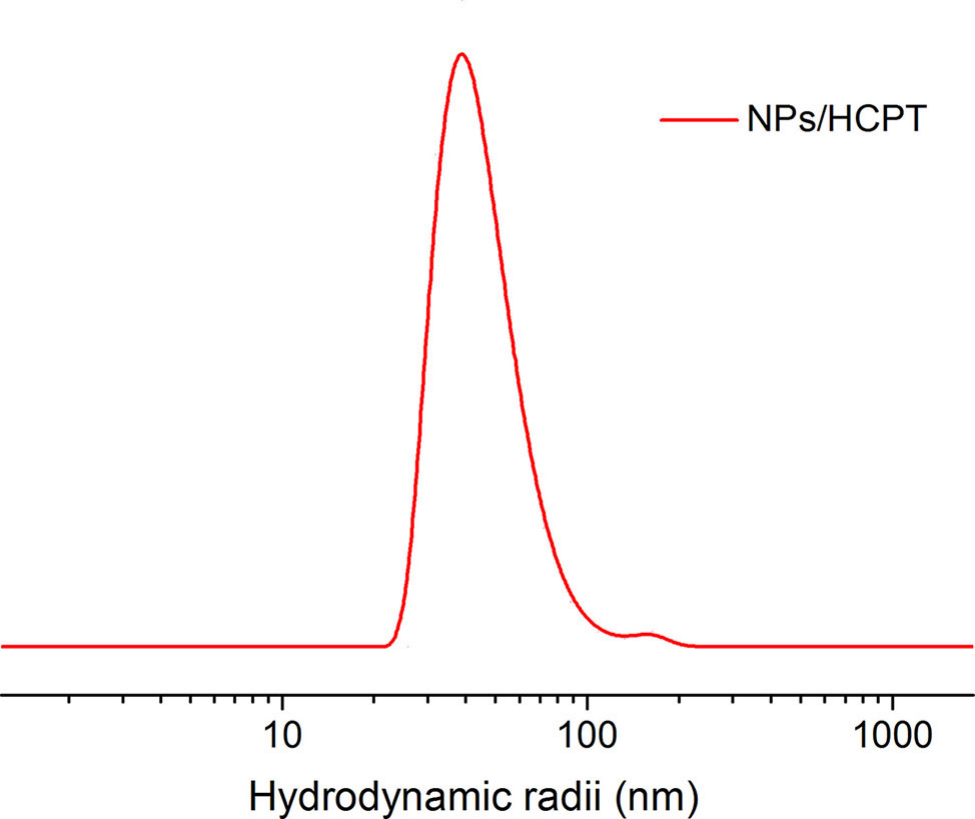
Hydrodynamic radii of NPs/10-Hydroxycamptothecin (HCPT).

The drug-loaded NPs/HCPT was fabricated by dialysis method, which was shown in **Figure 1**. Electrostatic forces may be the most important reason for drug encapsulation (Zhang et al., 2018). The simple preparation method predicted its good practicality. The average DLC of NPs/HCPT was 18.3 ± 0.9 wt.%. The DLE of NPs/HCPT was determined up to 80.2 ±1.5 wt.% (**Table 1**). The resulting NPs/HCPT exhibited an ideal diameter of 114.6 ± 4.1 nm, which was determined by DLS (**Figure 2**). The average diameter of NPs/HCPT ranged from 10 to 200 nm, resulting in the optimal tumor accumulation due to the enhanced permeability and retention (EPR) effect (Blanco et al., 2015). Furthermore, the *zeta* potential of NPs/HCPT was approximately 15.9 ± 2.4 mV (**Table 1**). A positive surface charge implied an enhanced interaction with the cell membranes, which was essential for increasing cell uptake and membrane permeation (Todorova et al., 2014). Apparently, NPs/HCPT is an excellent drug delivery platform due to the attractive electrostatic forces between the NPs/HCPT and biomembranes.

**Table 1:** Characterizations of NPs/10-Hydroxycamptothecin (HCPT).

Diameter (nm)	DLC (wt.%)	DLE (wt.%)	*zeta* Potential (mV)
114.6 ± 4.1	18.3 ± 0.9	80.2 ± 1.5	15.9 ± 2.4

### In Vitro HCPT Release

**Figure 3:**
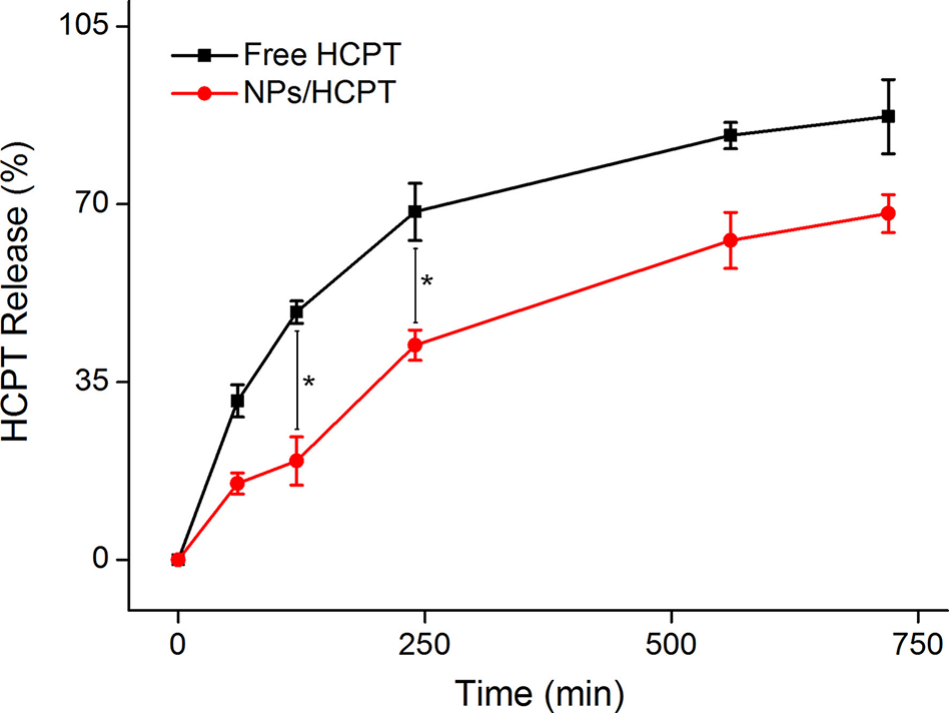
*In vitro* release profiles of 10-Hydroxycamptothecin (HCPT) from NPs/HCPT. Data are presented as mean ± SD (*n* = 2; *P < 0.05).

The *in vitro* HCPT release kinetics from the NPs/HCPT was shown in **Figure 3**, which was evaluated in PBS (pH 7.4, containing 0.1% Tween-80 (w/v)). Both free HCPT and NPs/HCPT displayed sustained release patterns. During the release process, free HCPT exhibited a very rapid release rate. However, the release of HCPT from the NPs/HCPT was decreased due to the encapsulation of CS. The prolonged and sustained drug release of NPs/HCPT indicated a relatively high level of HCPT concentration within cells, which was the key to enhance the anti-tumor activity *in vitro* and *in vivo*.

### Cell Uptake and Intracellular Distribution of NPs/HCPT

**Figure 4:**
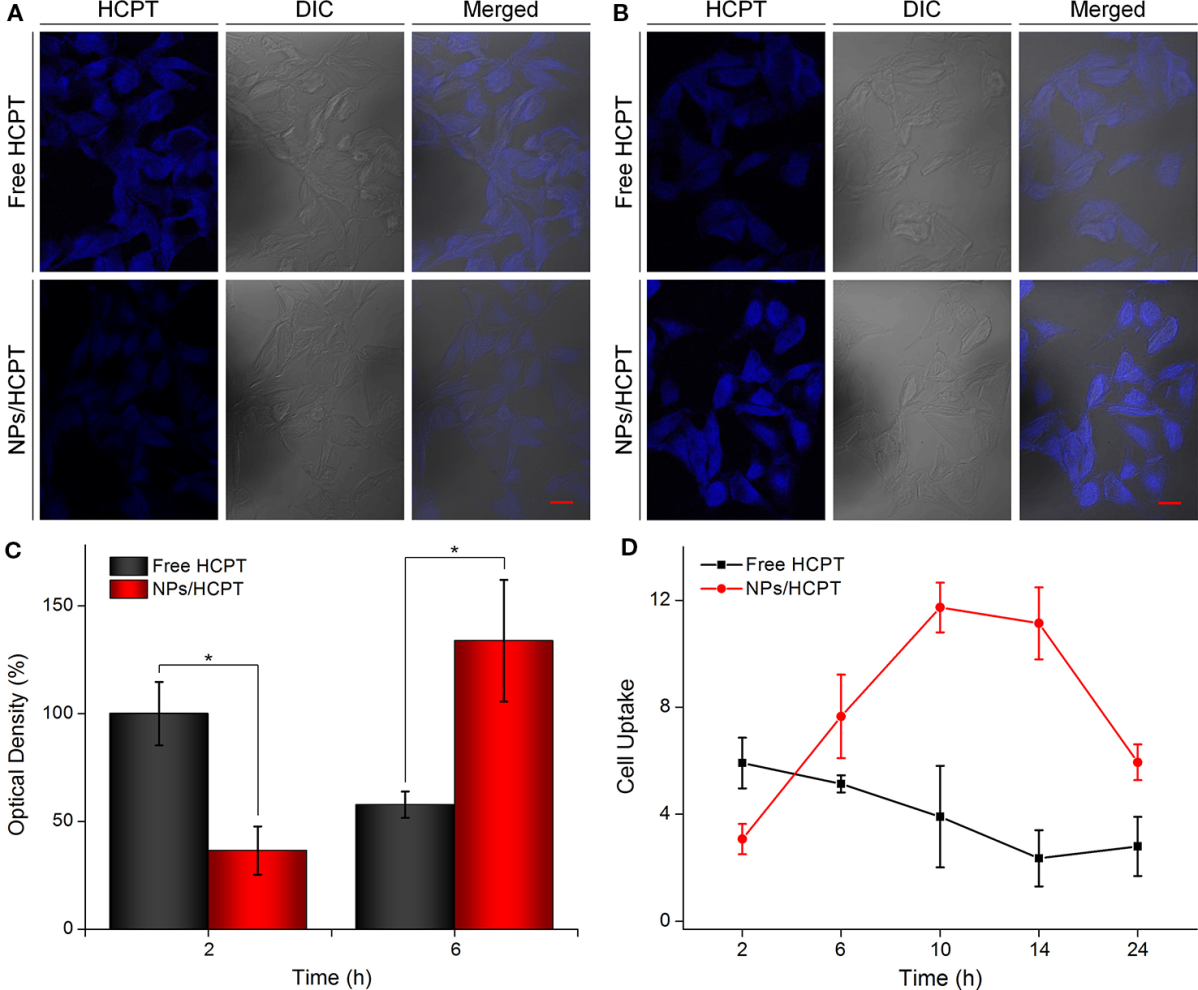
Cell uptake and intracellular distribution of NPs/10-Hydroxycamptothecin (HCPT). **(A)** Confocal microscopy images of B16F10 cells after incubation with free HCPT or NPs/HCPT for 2 h (scale bar, 20 μm). **(B)** Confocal microscopy images of B16F10 cells after incubation with free HCPT or NPs/HCPT for 6 h (scale bar, 20 μm). **(C)** Quantitative analysis of optical density of HCPT fluorescence intensity after 2 h or 6 h of treatment with free HCPT or NPs/HCPT. **(D)** Cell uptake of B16F10 cells treated with free HCPT or NPs/HCPT at a HCPT concentration of 1.5 μg ml^−1^. Data are presented as mean ± SD (*n* = 3; *P < 0.05).

NPs/HCPT treated B16F10 cells were visualized under CLSM to assess cell uptake of NPs/HCPT. Free HCPT treated cells revealed higher intracellular HCPT accumulation after 2 h of incubation, as compared to NPs/HCPT, which was depicted in **Figure 4A**. This phenomenon was due to the different ways in which drugs were transported into cells. It is reported that small molecules such as HCPT enter cells through simple diffusion (Xu et al., 2015; Zhao et al., 2017), while nanoparticles may be internalized by cells through endocytosis (Guo et al., 2017). However, with the incubation time prolonged to 6 h, the accumulation of free HCPT within B16F10 cells reduced, while the accumulation of NPs/HCPT within B16F10 cells increased significantly (**Figure 4B**). The relative optical density of intracellular HCPT was quantified with ImageJ software (National Institutes of Health, Bethesda, Maryland, USA). The HCPT fluorescence of free HCPT was 2.7 times higher than that of NPs/HCPT at 2 h. Conversely, the HCPT fluorescence of NPs/HCPT was 2.3 times higher than that of free HCPT at 6 h, which is depicted in **Figure 4C**. For further quantitative confirmation, the cell uptake of NPs/HCPT at different time points was performed by a microplate reader. As depicted in **Figure 4D**, the cell uptake of free HCPT decreased with time, while the internalization of NPs/HCPT increased first and then decreased, reaching a peak value at 10 h. The accumulation of NPs/HCPT in cells increased with time, suggesting that NPs/HCPT could be actively internalized by B16F10 cells in a time-dependent manner.

### NPs/HCPT Decreased the Cell Viability of Melanoma Cell Lines Dependent on Time and Concentration

**Figure 5:**
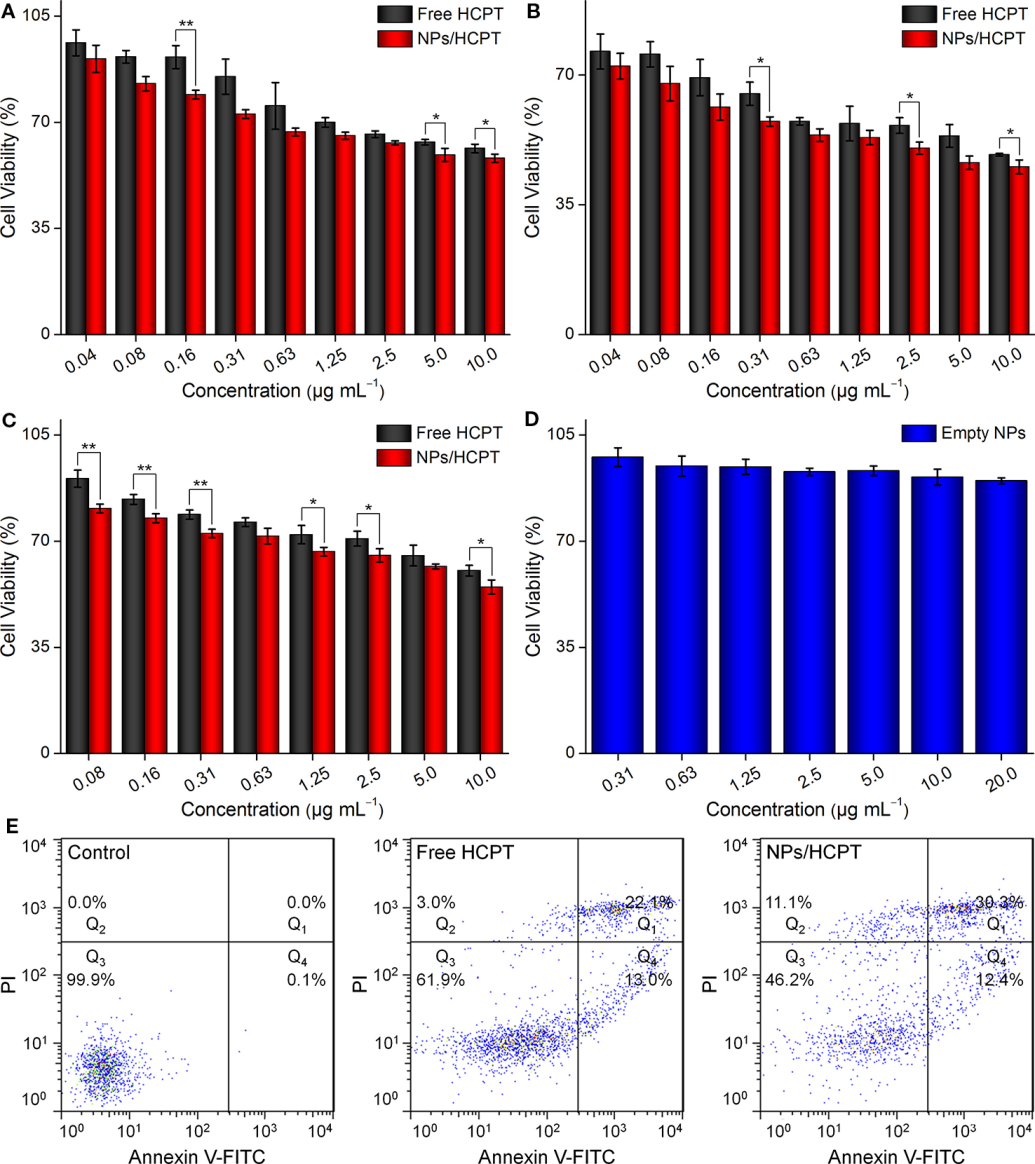
Cell cytotoxicity. **(A)** Viabilities of B16F10 cells incubated with different concentrations of free 10-Hydroxycamptothecin (HCPT) or NPs/HCPT for 24 h. **(B)** Viabilities of B16F10 cells incubated with different concentrations of free HCPT or NPs/HCPT for 48 h. **(C)** Viabilities of B16F1 cells incubated with different concentrations of free HCPT or NPs/HCPT for 24 h. **(D)** Viabilities of B16F1 cells incubated with empty NPs for 24 h. **(E)** The percentage of apoptotic cells was analyzed by flow cytometry (FCM) with Annexin V-FITC and Propidium iodide (PI) staining after different treatments for 24 h. Data are presented as mean ± SD (*n* = 3; *P < 0.05, **P < 0.01).

The inhibitory effect of NPs/HCPT on the cell viability of B16F10 was evaluated using the MTT assay. The results showed that NPs/HCPT inhibited the growth of B16F10 cells in a time and concentration-dependent manner. Concretely, the inhibition percentage of B16F10 cells in different conditions was displayed in **Figures 5A, B**. It was observed that there were obvious differences in cell viability between the two groups after incubation with different concentrations of free HCPT or NPs/HCPT for 24 and 48 h. Moreover, the viability of B16F10 cells treated with NPs/HCPT was found to be lower than that of free HCPT after 24 h and 48 h of incubation. By a non-linear regression analysis, it was calculated that NPs/HCPT showed a half maximal inhibitory concentration (IC_50_) of 2.4 μg ml^−1^ in B16F10 cells at 48 h. In comparison, the IC_50_ of free HCPT was 6.7 μg ml^−1^ at 48 h. At the same treatment time of 24 h, free HCPT and NPs/HCPT demonstrated well pronounced cytotoxicity against B16F1, which was similar to that on B16F10 cells at concentrations (**Figure 5C**). The empty NPs did not show significant cytotoxic effect in B16F1 after 24 h exposure, which was displayed in **Figure 5D**. The apparently higher cytotoxicity of NPs/HCPT was directly attributed to the increase of intracellular HCPT concentration, which was due to the positive surface of NPs/HCPT enhanced cells internalization through endocytosis (Kim et al., 2010; Chen et al., 2018). NPs/HCPT is superior to free HCPT in inhibiting tumor cells, suggesting that CS as a drug release platform can improve the cytotoxicity of HCPT.

### NPs/HCPT Induced Apoptosis in B16F10 Cells

Concerning the effect of NPs/HCPT on B16F10 cell viability, we speculated whether the reduced cell viability of B16F10 was the result of apoptotic cell death induced by NPs/HCPT. Thus, the apoptotic percentage of B16F10 cells induced by 3.0 μg ml^−1^ of free HCPT or NPs/HCPT was evaluated by Annexin V-FITC/PI double staining and FCM analysis. As showed in **Figure 5E**, an even higher percentage of apoptotic cells induced by NPs/HCPT were observed, as compared to that of free HCPT. These results indicated that NPs/HCPT not only possessed the function of inhibiting tumor, but also performed well in the delivery of HCPT into cancer cells. Therefore, as a nanoscale carrier for HCPT, NPs/HCPT has the potential to become an excellent drug delivery system for cancer therapy.

### In Vivo Antitumor Activity of NPs/HCPT

**Figure 6:**
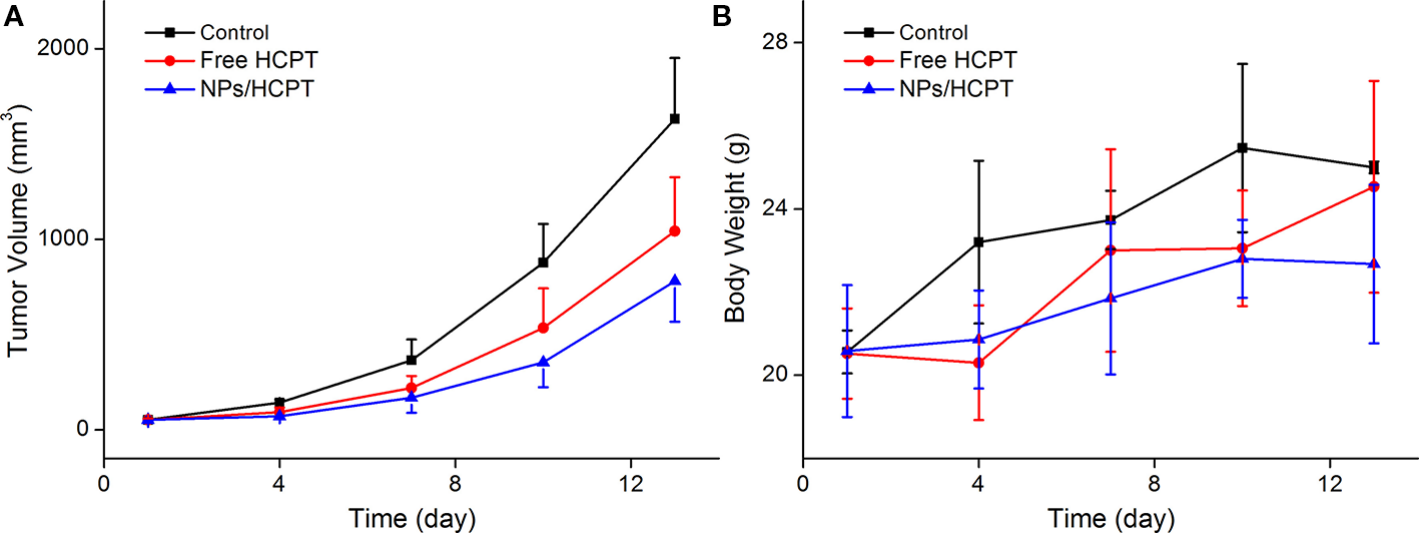
Tumor inhibition *in vivo*. **(A)** Time profiles of tumor growth in different mice after four treatments of phosphate-buffered saline (PBS), free 10-Hydroxycamptothecin (HCPT), or NPs/HCPT. **(B)** Body weight of tumor-bearing mice during the process of treatment. Data are presented as mean ± SD (*n* = 5).

The *in vivo* antitumor activity of NPs/HCPT was further confirmed in tumor-bearing mice. As exhibited in **Figure 6A**, free HCPT and NPs/HCPT inhibited the progression of tumors as compared with PBS. Moreover, NPs/HCPT showed a higher antitumor efficacy than that of free HCPT. All the tumor-bearing mice treated with NPs/HCPT showed no distinct difference in body weight during the process of the treatment, suggesting negligible toxicity (**Figure 6B**). The results indicated that NPs/HCPT exhibited an advantage of biological safety and excellent antitumor activity.

## Conclusions

In this work, cationic CS-based nanoparticles were prepared to enhance tumor penetration capability of HCPT for the potential chemotherapy of melanoma. The aqueous dispersibility of hydrophobic HCPT was significantly improved. The resulting NPs/HCPT exhibited an ideal diameter of 114.6 ± 4.1 nm, which was the optimal tumor accumulation size for the EPR effect. The positive surface charge and sustained release behavior ensured a high intracellular drug concentration. The MTT assay and FCM analysis suggested that NPs/HCPT exhibited a greater cytotoxicity in comparison to free HCPT. Furthermore, NPs/HCPT significantly inhibited the progression of tumors in animal models. These investigations implied that NPs/HCPT could be effectively applied to improve the chemotherapeutic effect of melanoma.

## Data Availability Statement

All datasets generated for this study are included in the article.

## Author Contributions

CW and YH conceived the idea. HG carried out the experiments. FL, HQ, and SQ took part in the experiments and the discussion of the results. HG, JL, and FL drafted the manuscript. All authors read and approved the final manuscript.

## Funding

The study was financially supported by the Science and Technology Development Program of Jilin Province (Nos. 20180101167JC).

## Conflict of Interest

The authors declare that the research was conducted in the absence of any commercial or financial relationships that could be construed as a potential conflict of interest.

## References

[B1] Al-Batran S. E. Homann N. Pauligk C. Goetze T. O. Meiler J. Kasper S. (2019). Perioperative chemotherapy with fluorouracil plus leucovorin, oxaliplatin, and docetaxel versus fluorouracil or capecitabine plus cisplatin and epirubicin for locally advanced, resectable gastric or gastro-oesophageal junction adenocarcinoma (FLOT4): a randomised, phase 2/3 trial. Lancet 393 (10184), 1948–1957. 10.1016/S0140-6736(18)32557-1 30982686

[B2] Blanco E. Shen H. Ferrari M. (2015). Principles of nanoparticle design for overcoming biological barriers to drug delivery. Nat. Biotechnol. 33 (9), 941–951. 10.1038/nbt.3330 26348965 PMC4978509

[B3] Bray F. Ferlay J. Soerjomataram I. Siegel R. L. Torre L. A. Jemal A. (2018). Global cancer statistics 2018: GLOBOCAN estimates of incidence and mortality worldwide for 36 cancers in 185 countries. CA Cancer J. Clin. 68 (6), 394–424. 10.3322/caac.21492 30207593

[B4] Chen F. Ma K. Madajewski B. Zhuang L. Zhang L. Rickert K. (2018). Ultrasmall targeted nanoparticles with engineered antibody fragments for imaging detection of HER2-overexpressing breast cancer. Nat. Commun. 9 (1), 4141. 10.1038/s41467-018-06271-5 30297810 PMC6175906

[B5] Danhier F. (2016). To exploit the tumor microenvironment: Since the EPR effect fails in the clinic, what is the future of nanomedicine? J. Control Release 244 (Pt A), 108–121. 10.1016/j.jconrel.2016.11.015 27871992

[B6] Fan W. Yung B. Huang P. Chen X. (2017). Nanotechnology for Multimodal Synergistic Cancer Therapy. Chem. Rev. 117 (22), 13566–13638. 10.1021/acs.chemrev.7b00258 29048884

[B7] Guo H. Xu W. Chen J. Yan L. Ding J. Hou Y. (2017). Positively charged polypeptide nanogel enhances mucoadhesion and penetrability of 10-hydroxycamptothecin in orthotopic bladder carcinoma. J. Control Release 259, 136–148. 10.1016/j.jconrel.2016.12.041 28062300

[B8] Guo H. Li F. Xu W. Chen J. Hou Y. Wang C. (2018). Mucoadhesive Cationic Polypeptide Nanogel with Enhanced Penetration for Efficient Intravesical Chemotherapy of Bladder Cancer. Adv. Sci. (Weinh) 5 (6), 1800004. 10.1002/advs.201800004 29938183 PMC6010003

[B9] Jia X. Zhang Y. Zou Y. Wang Y. Niu D. He Q. (2018). Dual Intratumoral Redox/Enzyme-Responsive NO-Releasing Nanomedicine for the Specific, High-Efficacy, and Low-Toxic Cancer Therapy. Adv. Mater. 30 (30), e1704490. 10.1002/adma.201704490 29889325

[B10] Kim B. Han G. Toley B. J. Kim C. K. Rotello V. M. Forbes N. S. (2010). Tuning payload delivery in tumour cylindroids using gold nanoparticles. Nat. Nanotechnol. 5 (6), 465–472. 10.1038/nnano.2010.58 20383126 PMC2881185

[B11] Kwak S. Y. Lew T. T. S. Sweeney C. J. Koman V. B. Wong M. H. Bohmert-Tatarev K. (2019). Chloroplast-selective gene delivery and expression in planta using chitosan-complexed single-walled carbon nanotube carriers. Nat. Nanotechnol. 14 (5), 447–455. 10.1038/s41565-019-0375-4 30804482

[B12] Li J. Xu W. Li D. Liu T. Zhang Y. S. Ding J. (2018). Locally Deployable Nanofiber Patch for Sequential Drug Delivery in Treatment of Primary and Advanced Orthotopic Hepatomas. ACS Nano 12 (7), 6685–6699. 10.1021/acsnano.8b01729 29874035

[B13] Nezakati T. Seifalian A. Tan A. Seifalian A. M. (2018). Conductive Polymers: Opportunities and Challenges in Biomedical Applications. Chem. Rev. 118 (14), 6766–6843. 10.1021/acs.chemrev.6b00275 29969244

[B14] Sheng J. Han L. Qin J. Ru G. Li R. Wu L. (2015). N-trimethyl chitosan chloride-coated PLGA nanoparticles overcoming multiple barriers to oral insulin absorption. ACS Appl. Mater. Interf. 7 (28), 15430–15441. 10.1021/acsami.5b03555 26111015

[B15] Todorova N. Chiappini C. Mager M. Simona B. Patel I. I. Stevens M. M. (2014). Surface presentation of functional peptides in solution determines cell internalization efficiency of TAT conjugated nanoparticles. Nano Lett. 14 (9), 5229–5237. 10.1021/nl5021848 25157643 PMC5408925

[B16] Wei W. Yue Z. G. Qu J. B. Yue H. Su Z. G. Ma G. H. (2010). Galactosylated nanocrystallites of insoluble anticancer drug for liver-targeting therapy: an in vitro evaluation. Nanomed. (Lond.) 5 (4), 589–596. 10.2217/nnm.10.27 20528454

[B17] Xu W. Ding J. Xiao C. Li L. Zhuang X. Chen X. (2015). Versatile preparation of intracellular-acidity-sensitive oxime-linked polysaccharide-doxorubicin conjugate for malignancy therapeutic. Biomaterials 54, 72–86. 10.1016/j.biomaterials.2015.03.021 25907041

[B18] Zhang Y. Cai L. Li D. Lao Y.-H. Liu D. Li M. (2018). Tumor microenvironment-responsive hyaluronate-calcium carbonate hybrid nanoparticle enables effective chemotherapy for primary and advanced osteosarcomas. Nano Res. 11 (9), 4806–4822. 10.1007/s12274-018-2066-0

[B19] Zhao K. Li D. Xu W. Ding J. Jiang W. Li M. (2017). Targeted hydroxyethyl starch prodrug for inhibiting the growth and metastasis of prostate cancer. Biomaterials 116, 82–94. 10.1016/j.biomaterials.2016.11.030 27914269

[B20] Zhao X. Guo B. Wu H. Liang Y. Ma P. X. (2018). Injectable antibacterial conductive nanocomposite cryogels with rapid shape recovery for noncompressible hemorrhage and wound healing. Nat. Commun. 9 (1), 2784. 10.1038/s41467-018-04998-9 30018305 PMC6050275

